# Variable coastal hypoxia exposure and drivers across the southern California Current

**DOI:** 10.1038/s41598-021-89928-4

**Published:** 2021-05-25

**Authors:** Natalie H. N. Low, Fiorenza Micheli, Juan Domingo Aguilar, Daniel Romero Arce, Charles A. Boch, Juan Carlos Bonilla, Miguel Ángel Bracamontes, Giulio De Leo, Eduardo Diaz, Eduardo Enríquez, Arturo Hernandez, Ramón Martinez, Ramon Mendoza, Claudia Miranda, Stephen Monismith, Mario Ramade, Laura Rogers-Bennett, Alfonso Romero, Carmina Salinas, Alexandra E. Smith, Jorge Torre, Gustavo Villavicencio, C. Brock Woodson

**Affiliations:** 1 Hopkins Marine Station, Stanford University, Pacific Grove, CA USA; 2 Monterey Bay Aquarium Research Institute, Moss Landing, CA USA; 3 Stanford Center for Ocean Solutions, Stanford University, Pacific Grove, CA USA; 4 Sociedad Cooperativa de Producción Pesquera Progreso, La Bocana, Baja California Sur México; 5 Sociedad Cooperativa de Producción Pesquera Pescadores Nacionales de Abulón, Isla Cedros, Baja California Sur México; 6 Southwest Fisheries Science Center, National Oceanic and Atmospheric Administration, San Diego, CA USA; 7 Sociedad Cooperativa de Producción Pesquera La Purísima, Bahía Tortugas, Baja California Sur México; 8 Sociedad Cooperativa de Producción Pesquera Ensenada, El Rosario, Baja California México; 9 Comunidad y Biodiversidad, La Paz, Baja California Sur México; 10 Sociedad Cooperativa de Producción Pesquera Punta Abreojos, Punta Abreojos, Baja California Sur México; 11 Sociedad Cooperativa de Producción Pesquera Buzos y Pescadores de la Baja California, Isla Natividad, Baja California Sur México; 12 Sociedad Cooperativa de Producción Pesquera Bahía Tortugas, Bahía Tortugas, Baja California Sur México; 13 Sociedad Cooperativa de Producción Pesquera California San Ignacio, Bahía Asunción, Baja California Sur México; 14 Department of Civil and Environmental Engineering, Stanford University, Stanford, CA 94305 USA; 15 Federación Regional de Sociedades Cooperativas de la Industria Pesquera Baja California, Ensenada, Baja California México; 16 Coastal Marine Science Institute, Karen C. Drayer Wildlife Health Center, University of California Davis, Davis, CA USA; 17 California Department of Fish and Wildlife, Bodega Marine Laboratory, Bodega Bay, CA USA; 18 Sociedad Cooperativa de Producción Pesquera Emancipación, Bahía Tortugas, Baja California Sur México; 19 Scoot Science, Santa Cruz, CA USA; 20 Sociedad Cooperativa de Producción Pesquera Leyes de Reforma, Bahía Asunción, Baja California Sur México; 21 COBIA Lab, University of Georgia, Athens, GA USA

**Keywords:** Ocean sciences, Climate-change ecology, Ecophysiology

## Abstract

Declining oxygen is one of the most drastic changes in the ocean, and this trend is expected to worsen under future climate change scenarios. Spatial variability in dissolved oxygen dynamics and hypoxia exposures can drive differences in vulnerabilities of coastal ecosystems and resources, but documentation of variability at regional scales is rare in open-coast systems. Using a regional collaborative network of dissolved oxygen and temperature sensors maintained by scientists and fishing cooperatives from California, USA, and Baja California, Mexico, we characterize spatial and temporal variability in dissolved oxygen and seawater temperature dynamics in kelp forest ecosystems across 13° of latitude in the productive California Current upwelling system. We find distinct latitudinal patterns of hypoxia exposure and evidence for upwelling and respiration as regional drivers of oxygen dynamics, as well as more localized effects. This regional and small-scale spatial variability in dissolved oxygen dynamics supports the use of adaptive management at local scales, and highlights the value of collaborative, large-scale coastal monitoring networks for informing effective adaptation strategies for coastal communities and fisheries in a changing climate.

## Introduction

Ocean deoxygenation is currently one of the most drastic changes occurring in marine ecosystems^1,2^. Coastal ecosystems are particularly susceptible, with declines in oxygen level occurring more rapidly along the coast compared to open ocean ecosystems^3,4^. Further, the intensity and frequency of deoxygenation is expected to worsen with climate change over time^5–9^. Oxygen levels in some coastal ecosystems may be nearing thresholds below which fisheries, biodiversity and ecosystems may collapse^1^ negatively impacting marine species, populations, ecosystems, and the services they provide humans. In coastal marine ecosystems, exposure to hypoxia (commonly defined as < 2 mg/L^10–13^, can cause direct mortality^14,15^ or severely impact feeding behavior, movement, growth, and reproductive processes, and reduce habitat quality for marine species^13,16–19^. These impacts can in turn scale up to impacts on populations, community composition, and fisheries^2,10,15,20^. Many of these impacts of hypoxia are exacerbated by interactions with other co-occurring stressors. In particular, warm temperatures can increase organisms’ vulnerability to hypoxia by enhancing their metabolic demand for oxygen^21–23^. Hypoxia can also increase vulnerability to the impacts of fishing, as when organisms fleeing from hypoxia aggregate at edges of low oxygen waters, and are targeted by fishers^24^.

Spatial and temporal patterns of variability in coastal hypoxia can influence their severity and impact on coastal ecosystems. Larger-scale seasonal drivers of hypoxia such as weather-driven nutrient loading, stratification, water temperatures, and upwelling patterns^6,10^ can interact with finer-scale transport processes such as tidal flushing and internal bores to generate complex patterns of temporal variability in dissolved oxygen^25,26^. These processes can also interact with the physical structure of nearshore habitats to produce local-scale spatial differences in the severity and temporal patterns of exposure to hypoxia^27–29^. For example, in the Monterey Bay region of California, USA, regional wind-driven upwelling transports cold, hypoxic deep water up onto the shelf, local internal bores move this hypoxia water into the shallow nearshore in distinct pulses, and where these internal bores surge and recede along local kelp forest rocky reef topography, hypoxic water can pool in benthic depressions to generate highly localized (< 10 m) spatial mosaics of hypoxic exposure^29,30^. Spatial variation in the severity and patterns of exposure to physiological stressors can provide natural refuges for organisms from large-scale climate impacts. Mobile organisms may be able to respond to spatiotemporal shifts in oxygen conditions by moving to escape hypoxia or even take advantage of periodic hypoxia to increase predation on less mobile, and therefore more vulnerable prey^31^ whereas sessile or sedentary organisms often benefit most from persistent refuges^32,33^. Identifying and harnessing such refuges, e.g., for the siting of seasonal or permanent fishing protections and restoration projects, has been suggested as a promising strategy for coastal communities and management agencies to implement climate change adaptation and conservation^34,35^. However, regional-scale analyses of dissolved oxygen dynamics are still uncommon, particularly in open-coast upwelling systems, and cross-shelf data on oxygen dynamics are not always particularly useful for the communities and marine resource managers whose interests lie mostly in shallow nearshore habitats. Continuous, high-resolution observations of dissolved oxygen conditions in coastal upwelling ecosystems, at depths of greatest interest to coastal managers and resource users, remain lacking at regional scales. Such data are available for the first time in the highly productive California Current upwelling region, thanks to a collaborative, participatory monitoring network of academic, government, and NGO scientists, as well as fishing cooperatives from California, USA, and Baja California, Mexico. Here we analyse a year-long dataset of dissolved oxygen and temperature data from kelp forest and rocky reef ecosystems from this regional monitoring network. We document spatial and temporal exposures to hypoxia at multiple locations, as well as substantial spatial heterogeneity and seasonal variability in dissolved oxygen and temperature dynamics. We highlight potential drivers and consequences of these spatial differences, opportunities for local adaptation, and the value of collaborative regional monitoring networks in the face of escalating exposure to environmental stressors.

## Materials and methods

**Figure 1:**
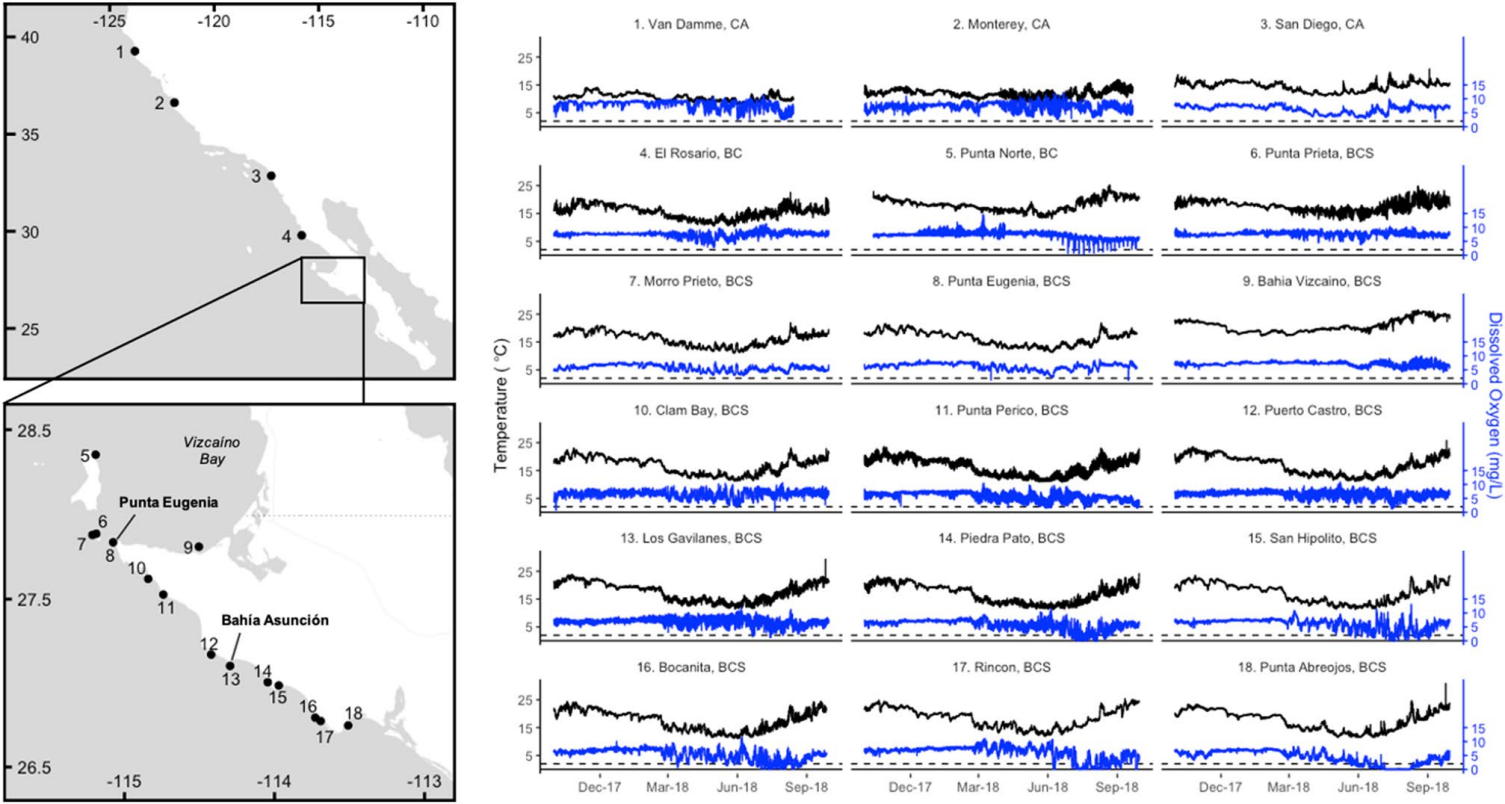
Temperature (black line) and dissolved oxygen (blue line) dynamics at 18 coastal sites in the southern California Current, over the 1-year period between October 2017 and September 2018. The dashed line represents the 2 mg/L threshold of hypoxia.

We analysed a year of time series data (October 2017–September 2018) for seawater temperature (°C) and dissolved oxygen (DO, mg/L) from the regional network of oceanographic sensors located at 18 sites in kelp forest/rocky reef ecosystems, spanning 13° of latitude from northern California, USA, to central Baja California, Mexico (Fig. 1, Table 1). Most of these sensors were sited, deployed, and maintained in partnership with local fishing cooperatives from the Federación Regional de Sociedades Cooperativas de la Industria Pesquera (FEDECOOP), the civil association Comunidad y Biodiversidad in Baja California, and the California Department of Fish and Wildlife. Deployment sites were all selected on the basis of being known suitable habitat for ecologically and economically important benthic invertebrate species, particularly red, green, and pink abalone (*Haliotis rufescens*, *H. fulgens*, and *H. corrugata*), which have previously suffered widespread mortality from hypoxic exposures at sites in this region (Isla Natividad^27,36^, La Bocana, Micheli et al., unpublished data). Sensors were located at depths of 6 to 15 m (Table 1), falling within the overlapping depth ranges of these abalone species (*H. rufescens*: low intertidal to 40 m; *H. fulgens*: low intertidal to 18 m, *H. corrugata*: 6–30 m^37^. Other co-occurring valuable benthic fisheries include spiny lobster (*Panulirus interruptus*), sea urchins (*Mesocentrotus franciscanus* and *Strongylocentrotus purpuratus*), sea cucumber (*Parastichopus parvimensis*) and turban snails (*Megastraea* spp.).

**Table 1:** Summary of sensor deployment sites.

	Site Name	Latitude	Longtitude	Depth (m)	Sensor Type
1-VD	Van Damme, CA	39.271833	− 123.79563	10	PME MiniDOT
2-MRY	Monterey, CA	36.621	− 121.899	17	GF Signet Resistance Thermometer, In Situ RDO Pro-X
3-SD	San Diego, CA	32.81320	− 117.29006	20	PME MiniDOT
4-SPF	El Rosario, BC	29.79099	− 115.80957	14	PME MiniDOT
5-PN	Punta Norte, BC	28.3532	− 115.19258	9	PME MiniDOT
6-PP	Punta Prieta, BCS	27.88691	− 115.18875	14	PME MiniDOT/ Seabird SBE 37
7-MP	Morro Prieto, BCS	27.87984	− 115.21378	15	PME MiniDOT/ Seabird SBE 37
8-EUG	Punta Eugenia, BCS	27.83632	− 115.07539	10	PME MiniDOT
9-VIZ	Bahia Vizcaino, BCS	27.8105	− 114.50412	10.5	PME MiniDOT
10-CB	Clam Bay, BCS	27.62015	− 114.84217	6	PME MiniDOT
11-PER	Punta Perico, BCS	27.5271	− 114.74084	13	PME MiniDOT
12-CAS	Puerto Castro, BCS	27.17093	− 114.42178	11	PME MiniDOT
13-GAV	Los Gavilanes, BCS	27.10345	− 114.2965	9	PME MiniDOT
14-PAT	Piedra Pato, BCS	27.00639	− 114.04489	14	PME MiniDOT
15-HIP	San Hipolito, BCS	26.987267	− 113.97182	7	PME MiniDOT
16-BCN	Bocanita, BCS	26.79495	− 113.72841	10	PME MiniDOT
17-RIN	Rincon, BCS	26.77482	− 113.69234	11	PME MiniDOT
18-ABR	Punta Abreojos, BCS	26.74856	− 113.50941	12	PME MiniDOT

For all sites except Monterey, temperature and DO were measured using autonomous sensors (PME MiniDOTs and/or Seabird SBE37-ODO, see Table 1) deployed within 1 m of the bottom, within or directly adjacent to kelp beds. In Baja California and Baja California Sur, sensor locations were selected by local fishing cooperatives based on their knowledge of present or past productive abalone fishing habitat. At these sites, sensors were deployed and maintained by divers from local fishing cooperatives, with the support and participation of academic and NGO scientists and staff. The Van Damme and San Diego sites correspond to locations where the California Department of Fish and Wildlife has conducted long-term abalone population monitoring. Sensors at these two sites were deployed and maintained by scientists and staff from the California Department of Fish and Wildlife. Sensors at all sites logged measurements at 10-min intervals with the exception of the Van Damme site, which logged measurements at 1-min intervals. At the Monterey site, temperature and DO were sampled from seawater drawn through the Monterey Bay Aquarium’s intake pipes, located at 17 m and adjacent to kelp forest habitat. Measurements were logged at intervals of 5 min using wired probes (GF Signet Resistance Thermometer for temperature, In Situ RDO Pro-X for DO) that were cleaned and calibrated regularly. More details of this site, including intake flow rates are available at^25^. At all other sites, sensors were cleaned and batteries changed at least once during the 12-month deployment.

To maintain identical sampling intervals across all sites, we filtered the time series data from the Van Damme and Monterey sites to obtain subsets of the data with 10-min intervals between measurements. We used the temperature and DO time series data to calculate summary statistics to characterize temperature and DO dynamics, as well as hypoxia exposures. We calculated mean and maximum temperature, minimum DO, coefficient of variation in DO, mean absolute rate of change in DO between adjacent data points, the number of exposures to hypoxic conditions (defined as an exposure to the widely-used 2 mg/L threshold for hypoxia^10–13^ for an hour or more), the mean duration of these hypoxic events, the mean duration between the end of a hypoxic event and the start of the next one (“return time”), for each monitored site. We calculated mean temperature and DO level for each day of the year and plotted these values on a temperature-DO biplot to visualize shifts in seawater characteristics throughout the year.

For sites that experienced hypoxia, we also took the number of hypoxic events observed for each month of the study and divided them by the total number of events observed at the site to calculate the proportion of hypoxic events occurring in each month to investigate potential seasonal patterns. To assess the potential role of upwelling as a driver of dissolved oxygen dynamics across the year, we calculated monthly correlation coefficients between temperature and dissolved oxygen at each site. We assessed timescales of variability for temperature and DO by calculating variance spectra for the time series data. Power spectra were computed using the Welch method with a Hamming window of 90 days and a 50% overlap. Spectra were then scaled to the variance in DO. Variances were then integrated across seasonal (0.01–0.05 cycles per day; cpd), synoptic (scales of mesoscale variability and atmospheric weather patterns, 0.1–0.5 cpd), diurnal (0.75–1.25 cpd), and semidiurnal (1.75–2.25 cpd) period bands, corresponding to 20–100 day, 2–10 day, ~ 24-h, and ~ 12-h cycles respectively.

## Results

**Figure 2:**
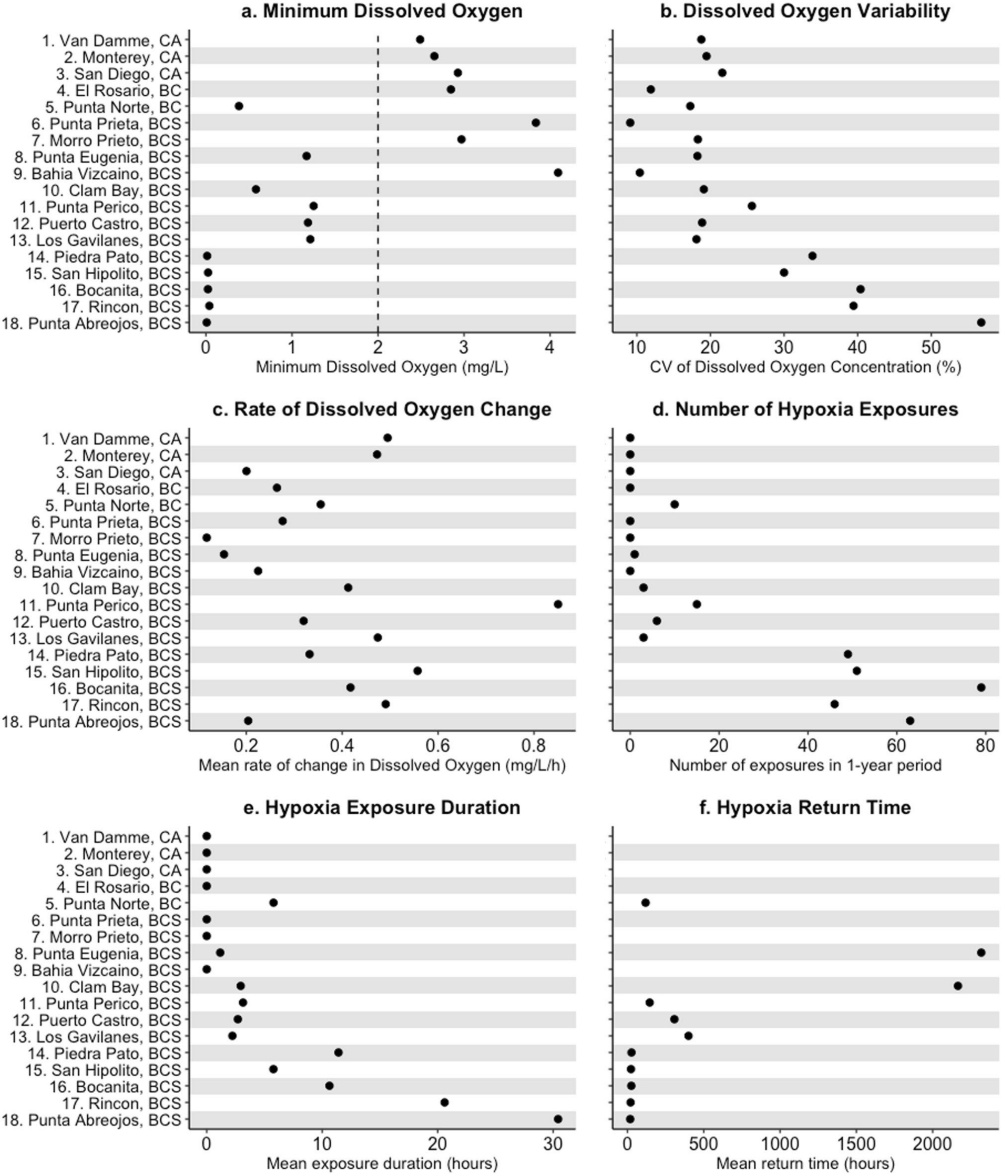
Summary metrics of dissolved oxygen (DO) variability at 18 sites in the southern California Current between October 2017 and September 2018: (**a**) minimum DO recorded during the year; (**b**) coefficient of variation in DO; (**c**) mean rate of change in DO; (**d**) total number of exposures to hypoxic conditions (≤ 2 mg/L); (**e**) mean duration of exposure to hypoxia; (**f**) mean return time between exposures to hypoxia. Sites are arranged by latitude from north to south. The dashed line indicates the 2 mg/L threshold for hypoxia.

We observed broad regional patterns in exposure to coastal hypoxia throughout the study region. Dissolved oxygen levels fell below the 2 mg/L threshold for hypoxia at all sites southwest of the Punta Eugenia headland on the Vizcaino peninsula, in Baja California Sur, Mexico (sites 8, 10–18), as well as one site north of this headland, Punta Norte (Fig. 2a). Severely hypoxic (< 0.05 mg/L) conditions were recorded in the five southernmost sites (sites 14–18), south of Bahía Asunción. These five sites also experienced the greatest oxygen variability (Fig. 2b), the highest number of hypoxic exposure events (Fig. 2d), the longest hypoxia exposure durations (Fig. 2e), and the shortest hypoxia return times of about a day or less, although they did not also experience distinctly faster rates of change compared to other sites. Among the other sites that experienced hypoxia, mean return times were on the order of months for Punta Eugenia and Clam Bay, the northernmost sites on the western Vizcaino peninsula, and 1–2 weeks for Punta Perico, Puerto Castro, and Los Gavilanes, the three sites in the central area of this peninsula (Fig. 2f). Mean return time was about a week for Punta Norte, the only site that experienced hypoxia north of the Punta Eugenia boundary.

Despite differences in sensor deployment depths among sites (6–20 m depth; Table 1), we did not see evidence that these differences impacted our detection of regional patterns of dissolved oxygen dynamics and exposures to hypoxia. Because exposures to low-oxygen water transported inshore by internal waves may be influenced by closer proximity to deeper offshore sources, we were concerned that deeper sensors would be more likely to detect lower dissolved oxygen concentrations as well as instances of hypoxia^34,35,38^. However, hypoxic events were also detected at sites with shallower sensors, and we only found a weak positive correlation between site sensor depth and the minimum dissolved oxygen recorded at the site (R^2^ = 0.21, *P* = 0.03). We did not find any correlations between sensor depth and the other dissolved oxygen summary variables (*P* > 0.27 for all variables).

**Figure 3:**
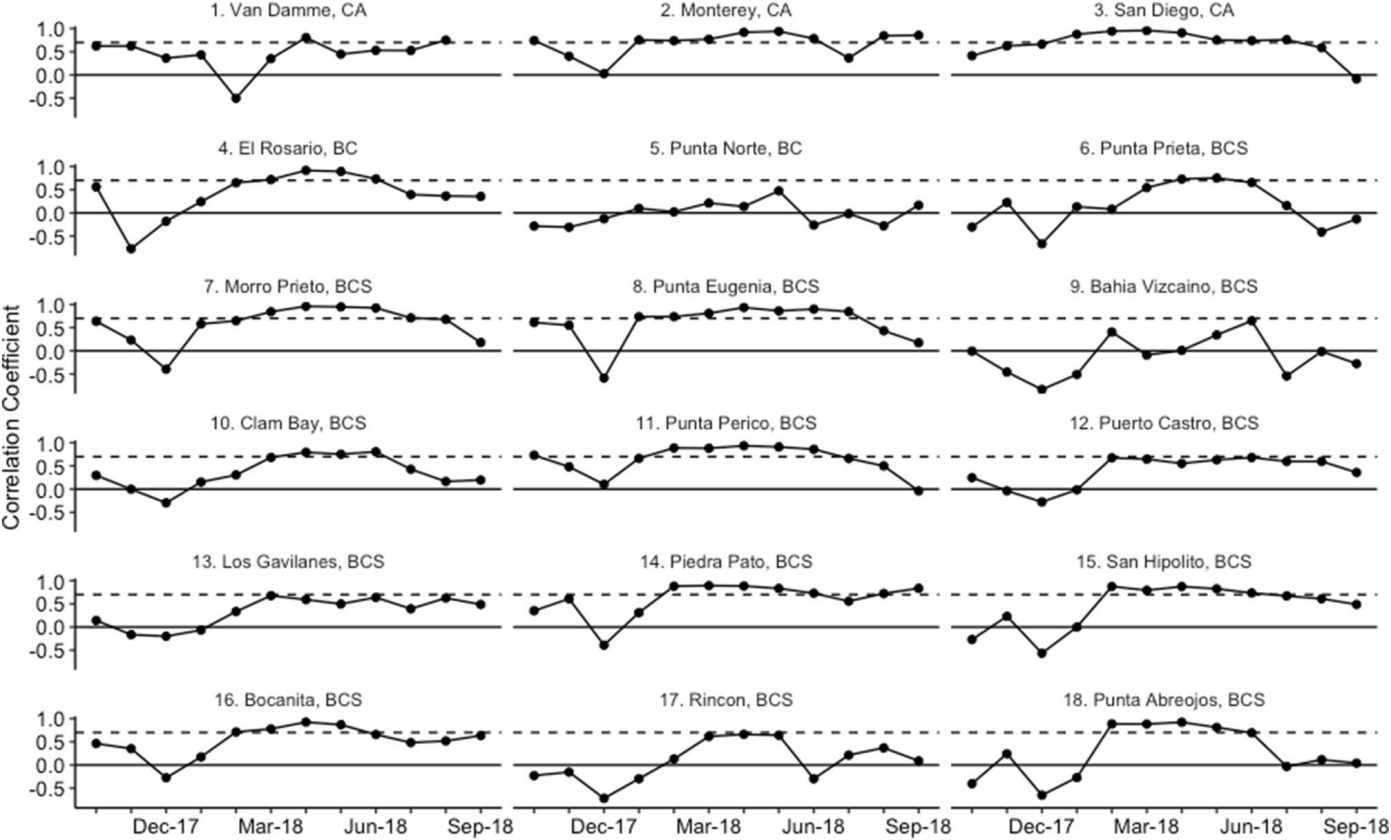
Monthly correlation values between temperature and dissolved oxygen for each site. The dashed line represents a correlation coefficient of 0.7, which is a significant positive correlation.

**Figure 4:**
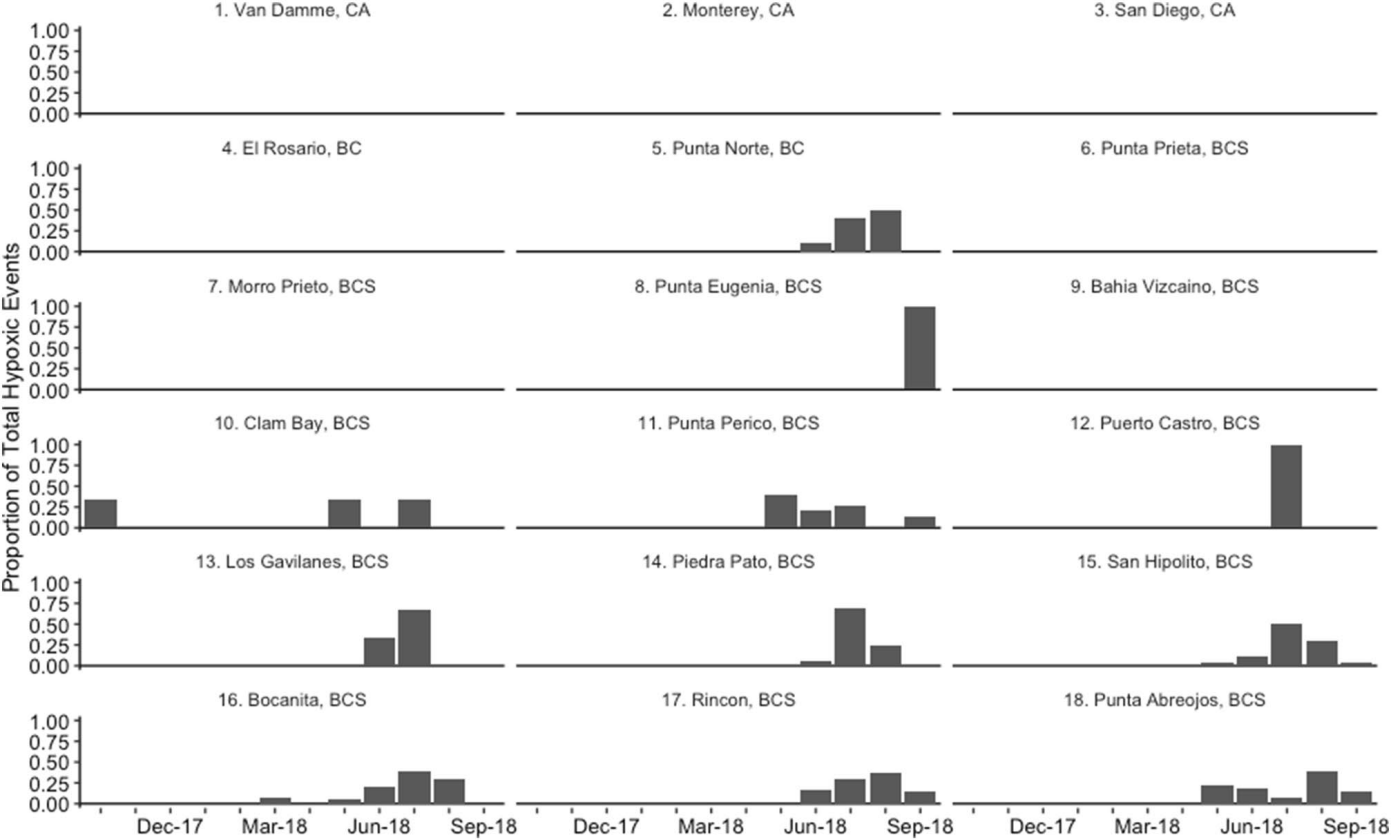
Proportion of total hypoxic (< 2 mg/L dissolved oxygen) events in each month, for each site.

We also observed broad-scale patterns for seasonality in temperature and dissolved oxygen dynamics. Across almost all sites, water temperatures cooled between October and May, during the winter months and upwelling season, and warmed again between June and September (Fig. 1, S1). At most sites, dissolved oxygen concentrations became much more temporally variable starting in the early spring, when dissolved oxygen levels experienced periodic decreases (Fig. 1). This led to overall decreases in mean values of dissolved oxygen between spring and summer (Fig. 1, S2). This springtime change was also associated with stronger positive correlations between water temperature and dissolved oxygen values (Fig. 3). The periodic decreases in dissolved oxygen led to their levels falling below hypoxic thresholds at Punta Norte and sites south of Punta Eugenia between May and October, but especially in the summer months of June to August (Figs. 1, 4, S2). In the two southernmost sites, Rincon and Punta Abreojos, water temperature and dissolved oxygen became uncorrelated during these months of hypoxic exposure (Fig. 3, S3d).

Exceptions to these broad seasonal trends occurred at sites most closely associated with the Vizcaíno Bay in Baja California (Punta Norte, Punta Prieta, and Bahía Vizcaíno; sites 5, 6, and 8), which were on average, 1–4 °C warmer than Morro Prieto and Punta Eugenia, the other two sites in the same area (Fig. 1, S1, S3). At Bahía Vizcaíno, water temperature warmed from January through the spring and summer, and dissolved oxygen levels stayed high with low variability during the spring (Fig. 1, S1, S2). Punta Norte and Punta Prieta showed temperature patterns similar to most other sites, but dissolved oxygen levels also remained high and less variable through the spring, relative to Morro Prieto and Punta Eugenia (Fig. 1, S2). Punta Norte experienced a series of regular low-oxygen events in the summer that occurred at water temperatures higher than the overall site average (Fig. 1, 3, S3). Water temperature and dissolved oxygen were generally not correlated at Punta Norte and Bahía Vizcaíno, but Punta Prieta showed seasonal correlation patterns similar to most other sites in the region (Fig. 3).

Variance spectra for dissolved oxygen showed distinct variance peaks at diurnal frequencies across all sites, and a semidiurnal peak was also present at many sites (Fig. S5). Integrated variance spectra showed that dominant timescales of variation were similar for temperature and dissolved oxygen dynamics, with the exception of Punta Norte and Bahía Vizcaíno, where temperature dynamics were dominated by synoptic (2–10 day) variation, while dissolved oxygen dynamics were dominated by diurnal (~ 24 h) variation. The integrated variance spectra also showed that there could be considerable among-site differences in dominant timescales of variance, even for nearby sites where broader exposure patterns seem relatively similar (i.e., Piedra Pato and San Hipolito (sites 14 and 15)).

## Discussion

Using data collected from our multi-national collaborative, participatory oceanographic monitoring network, we found evidence for broad regional and seasonal patterns in water temperature and dissolved oxygen dynamics and exposures to hypoxia, as well as local-scale differences, across sites spanning 13° of latitude in the southern California Current region. This is the first regional-scale analysis of nearshore dissolved oxygen dynamics in this highly variable and productive upwelling system. The observed patterns suggest the presence of multiple drivers of hypoxia, and that the vulnerability of coastal ecosystems and human communities to hypoxia is highly variable seasonally and across multiple spatial scales.

Coastal upwelling systems like the California Current region are often characterized by high variability in temperature, pH, and dissolved oxygen. Hypoxia in these systems can be driven by upwelling, alongshore transport of deep hypoxic water, shoaling internal waves, eutrophication, or a combination of these processes^15,25,26,39,40^. We found evidence for upwelling as a widespread regional driver of temperature and dissolved oxygen dynamics, starting in the spring, when most study sites showed decreases in water temperature, increased variability in dissolved oxygen levels, and tighter correlation between temperature and dissolved oxygen values. These springtime changes correspond to the period of time when upwelling is known to occur in the California Current system, transporting colder, less oxygenated water into shallower coastal habitats^25,41^. In addition to the region-scale influences of upwelling, we also found evidence for respiration-driven hypoxia at smaller spatial scales. We observed further declines in dissolved oxygen levels and longer, more intense exposures to hypoxia at the five sites south of Bahía Asunción in the summer months, accompanied by decreases in the correlation between temperature and dissolved oxygen. The effect was particularly pronounced at Rincon and Punta Abreojos, which experienced extensive exposures to near-anoxia. At these two southernmost sites, temperature and dissolved oxygen became decoupled in the summer months, suggesting that upwelling was no longer a key driver of oxygen dynamics. The intense hypoxia exposures at these southern sites corresponded spatially and temporally to reports of algal blooms (red tide) observations by the fishing cooperatives, which were also corroborated by red tide detection algorithms based on satellite images (Lee and Micheli, in prep). These cooperatives also reported mortalities of abalone and other benthic invertebrates in their fishing grounds during this period.

This combination of upwelling and respiration drivers cumulatively created a broad latitudinal pattern of hypoxia exposure, with hypoxia exposure mostly occurring only south of the Punta Eugenia headland, which is a well-known phylogeographic and biogeographic boundary for many taxa^42,43^. Among these affected sites, southern sites experienced more intense and extensive hypoxia exposures than northern sites. However, we also found evidence for smaller-scale differences in temperature and dissolved oxygen dynamics, suggesting that more localized drivers can alter the effects of these large-scale regional drivers. At Bahía Vizcaíno, Punta Norte, and to a lesser extent at Punta Prieta, dissolved oxygen levels remained high and less variable through the spring initiation of upwelling. These sites are within or oriented towards the Vizcaíno Bay rather than towards the Pacific Ocean and the prevailing wind direction, and are likely less influenced by wind-driven upwelling. They were consistently warmer than Morro Prieto and Punta Eugenia, the two westward-oriented sites in the same area. The general lack of correlation between temperature and dissolved oxygen, and the mismatch of dominant variation frequencies at Bahía Vizcaíno and Punta Norte also suggests that dynamics of the warmer, less-variable Vizcaíno Bay, rather than regional coastal upwelling processes, are the key drivers of conditions at these sites. Similarly, Punta Norte’s unique and highly localized series of intense exposures to hypoxia in the late summer are likely driven by processes other than upwelling. These hypoxic events were associated with high water temperatures and with decoupled temperature and dissolved oxygen dynamics, but were also not associated with algal blooms like the ones observed at southern sites. Finally, Punta Prieta appears to exhibit temperature and dissolved oxygen dynamics intermediate between the Vizcaíno Bay-oriented sites and the Pacific Ocean-oriented sites, likely due to the transport of water by strong tidal currents that circle the small island of Isla Natividad^34,35,44^. Because these multiple drivers of dissolved oxygen dynamics and hypoxia exposures act at different spatial scales and are expected to be impacted differently by global change^9,45,46^, understanding when and where the drivers differ is critical to anticipating the current and future occurrence and impacts of hypoxia.

Our data show that patterns of exposure and thus, potential ecological vulnerabilities to hypoxia, are highly variable on different spatial and temporal scales, and thus suggest that the valuable, hypoxia-vulnerable benthic fisheries in the region may be best managed at small spatial scales that match these differences in vulnerability. In Baja California, the involvement of local fishing cooperatives with ecological and oceanographic monitoring makes such small-scale adaptive management both a practical and desirable strategy^47^. These fishing cooperatives have already used data from this monitoring network to make management decisions. Cooperatives in the network independently access and visualize the temperature and oxygen data at their sites via an application developed by co-author A. Smith on the R Shiny platform^48,49^. Temperature data have been used to adjust the timing of fishing activities, such as by delaying the start of the fishing season after prolonged cold temperatures to allow more time for mollusk body conditions to recover after spawning. Oxygen data and documentation of hypoxia have motivated temporary closures of parts of the fishing grounds, to avoid adding fishing mortality to hypoxia induced mortality. Temperature and oxygen data have also informed the siting of mariculture, artificial reef and restoration projects in some cooperatives, by enabling identification of sites that are warmer and less prone to hypoxia. Creating similar local capacity for monitoring and for flexible local-scale adaptive management in other areas is crucial for more effective management and climate change adaptation across the California Current and other upwelling regions.

Our monitoring network has been successful at providing valuable information on spatial variability in hypoxia exposure. However, to evaluate spatial differences in vulnerability to hypoxia, spatial variation in dissolved oxygen dynamics must be integrated with the hypoxia sensitivity of species and processes in these ecosystems. In this study, we used the widely-used hypoxia threshold of 2 mg/L as a broad reference point. However, broadly-defined hypoxia thresholds may not be representative of tolerances for this particular system. There is a very limited amount of hypoxia threshold data available for species in the California Current system. Those that exist for benthic invertebrates suggest that both adult and juvenile life stages may be tolerant of oxygen levels much lower than 2 mg/L, but that physiological, behavioral, and ecological processes may be impacted at much higher levels of dissolved oxygen (e.g., 5.5 mg/L in sea urchins)^18,50,51^. These vulnerabilities also vary among taxa. For example, crustaceans tend to be sensitive to low oxygen conditions while mollusks and echinoderms tend to be more tolerant^13,50^. Species mobility will also have an impact on hypoxia vulnerability with sessile benthic organisms being less able to move away from localized hypoxia. A better understanding of species hypoxia tolerances in this and other systems will be crucial to assessing spatial differences in vulnerability to coastal hypoxia. Furthermore, understanding potential intraspecific differences in organism tolerances, especially relative to oceanographic and phylogeographic breaks (such as Punta Eugenia in our study region^42^), will be useful for assessing the potential for local adaptation.

This network of dissolved oxygen sensors has identified broad geographic trends and smaller-scale variability in hypoxia drivers and vulnerabilities, but further observations will be required to assess the longer-term persistence of these trends in an oceanographic system known for its high temporal variability and multi-year drivers, and under continuing climate change^41,46,52,53^. Previous multi-year monitoring has revealed localized high interannual variability and exposures to hypoxia at sites relatively unaffected during this study period^27,36^, and respiration-driven hypoxia associated with algal blooms has been reported at the northern California sites in the past, but was not observed from 2017 to 2018^54^. Although there is evidence that some spatial differences are maintained across years^34,35^, it is unclear if the broader, regional-scale patterns show similar levels of persistence. The longevity of this spatial mosaic will determine if and how local- and regional-scale variability in physiological stress can be useful in adaptation, management, and conservation.

Our collaborative, region-wide efforts so far have characterized hypoxia exposures in the important context of seawater temperature. However, dissolved oxygen dynamics needs to be monitored within an integrated, multi-stressor context. For example, pH is also known to correlate closely with temperature and dissolved oxygen during upwelling events^25,55^, and also to influence hypoxia tolerance^56^. Simultaneous exposures to oxygen, pH and temperature variation and extremes are likely to produce additive, synergistic or antagonistic effects on organisms^57,58^.

As the first characterization of regional-scale coastal hypoxia dynamics in the California Current upwelling system, this study provides a key step forward in demonstrating the complex, multiple-driver spatial mosaic of this increasingly important climate change stressor in coastal systems, and also highlights how knowledge co-production in multi-stakeholder groups can be instrumental in enabling such spatially extensive efforts. Our findings highlight the need for understanding this spatial and temporal variability to design realistic experiments and models to inform species and ecosystem vulnerabilities, and importantly, providing guidance for local and regional scale adaptive management. In particular, the identification of potential refuges from hypoxia provides promising opportunities for supporting local resilience through marine protected areas, restoration, and mariculture that leverage these microclimates^27,34,35^. The use of large-scale coastal monitoring networks, in partnership with local stakeholders and governments, will be invaluable for meeting this need in the face of regional and global ocean changes.

## Supplementary Information

Supplementary Information.
