# Supplementary material for: Variable coastal hypoxia exposure and drivers across the southern California Current
Source: Sci Rep. 2021 May 25;11:10929. doi: 10.1038/s41598-021-89928-4 (PMC8149850; doi:10.1038/s41598-021-89928-4)

**Supplementary material**

Figure S1. Mean daily temperatures at 18 coastal sites in the southern California Current from October 2017 to September 2018. Note the site-specific differences in y-axis scales; the figure is plotted to visualize seasonal changes at each site.


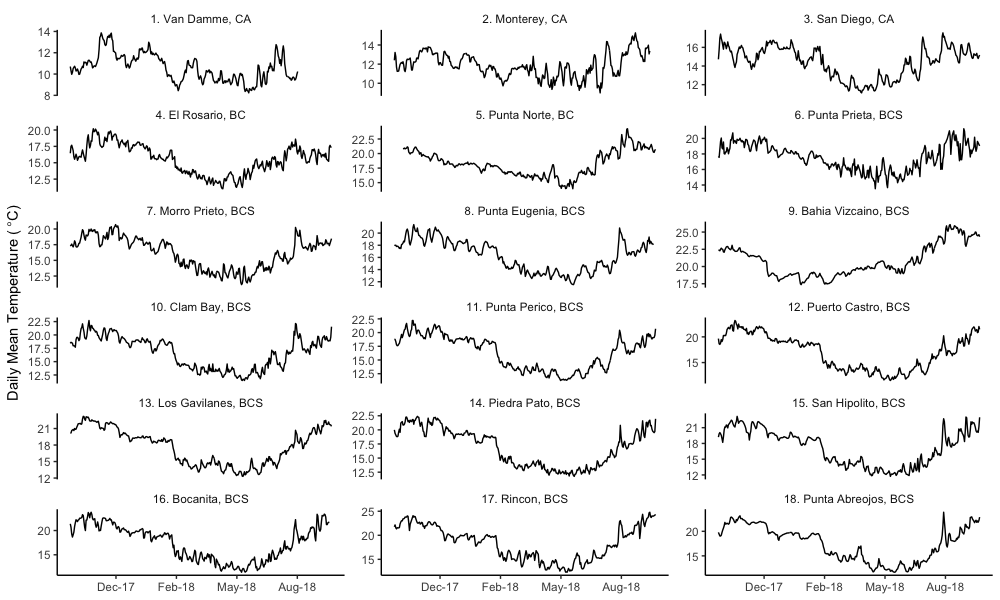


Figure S2. Mean daily dissolved oxygen concentrations at 18 coastal sites in the southern California Current from October 2017 to September 2018. The dashed line represents the 2 mg/L threshold for hypoxia.


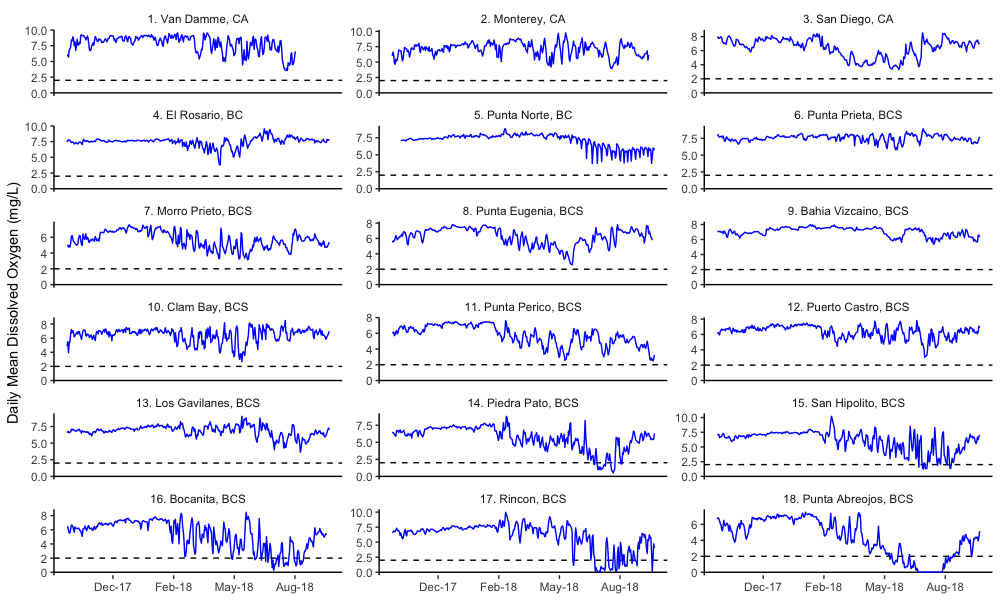


Figure S3 (a). Mean temperature; (b) maximum temperature; (c) mean temperature during hypoxic events; (d) mean correlation coefficient between temperature and dissolved oxygen during hypoxic events at 18 sites in the southern California Current during the year between October 2017 and September 2018. Sites are arranged by latitude from north to south. The dashed line represents a correlation coefficient of 0.7, which is a significant positive correlation.


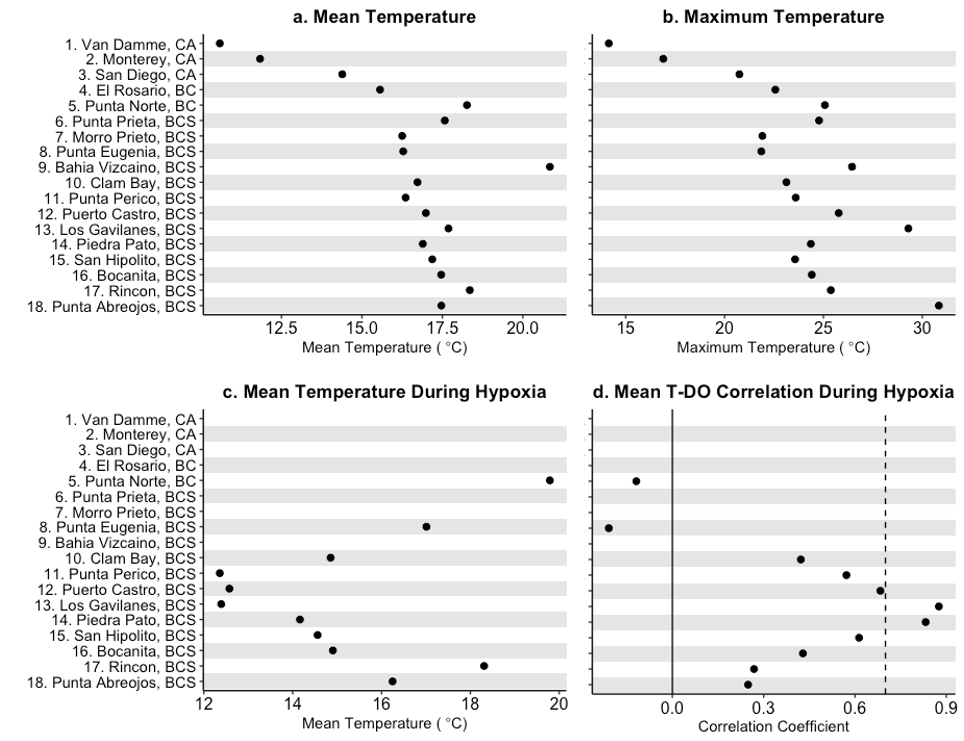


Figure S4. Variance spectra for temperature at 18 sites. Shaded bands (left to right) correspond to seasonal (0.01-0.05 cycles per day; cpd), synoptic (﻿scales of mesoscale variability and atmospheric weather patterns; 0.1-0.5 cpd), diurnal (0.75 – 1.25 cpd), and semidiurnal (1.75 – 2.25 cpd) periods. Note the site-specific differences in the range of variance (y-axis).


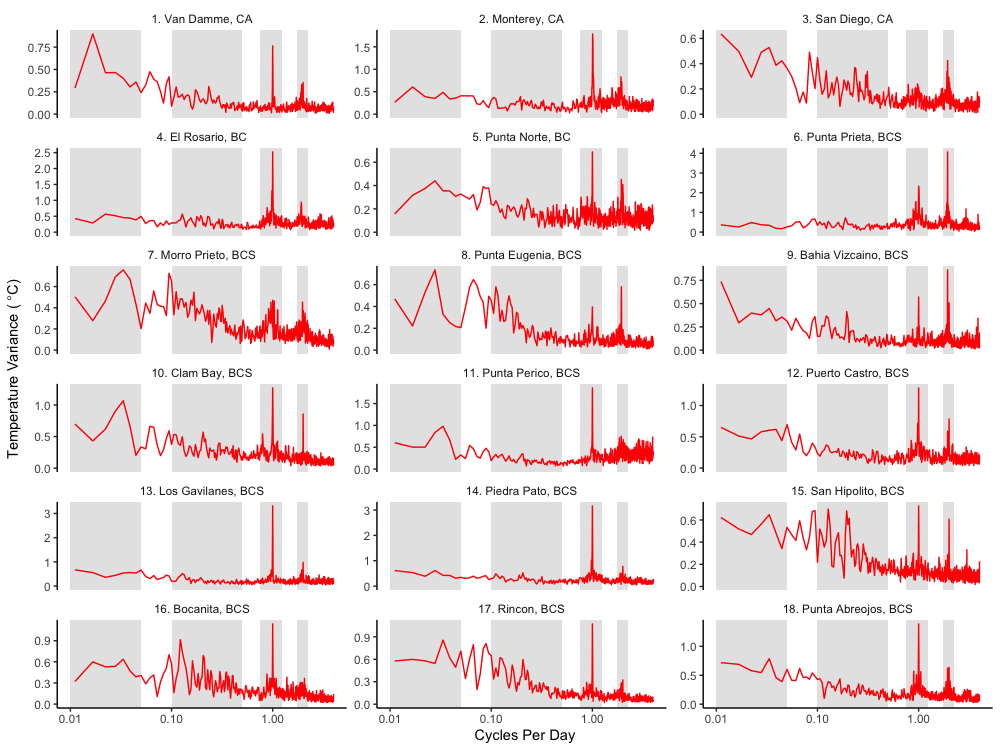


Figure S5. Variance spectra for dissolved oxygen at 18 sites. Shaded bands (left to right) correspond to seasonal (0.01-0.05 cycles per day; cpd), synoptic (﻿scales of mesoscale variability and atmospheric weather patterns; 0.1-0.5 cpd), diurnal (0.75 – 1.25 cpd), and semidiurnal (1.75 – 2.25 cpd) periods. Note the site-specific differences in the range of variance (y-axis).


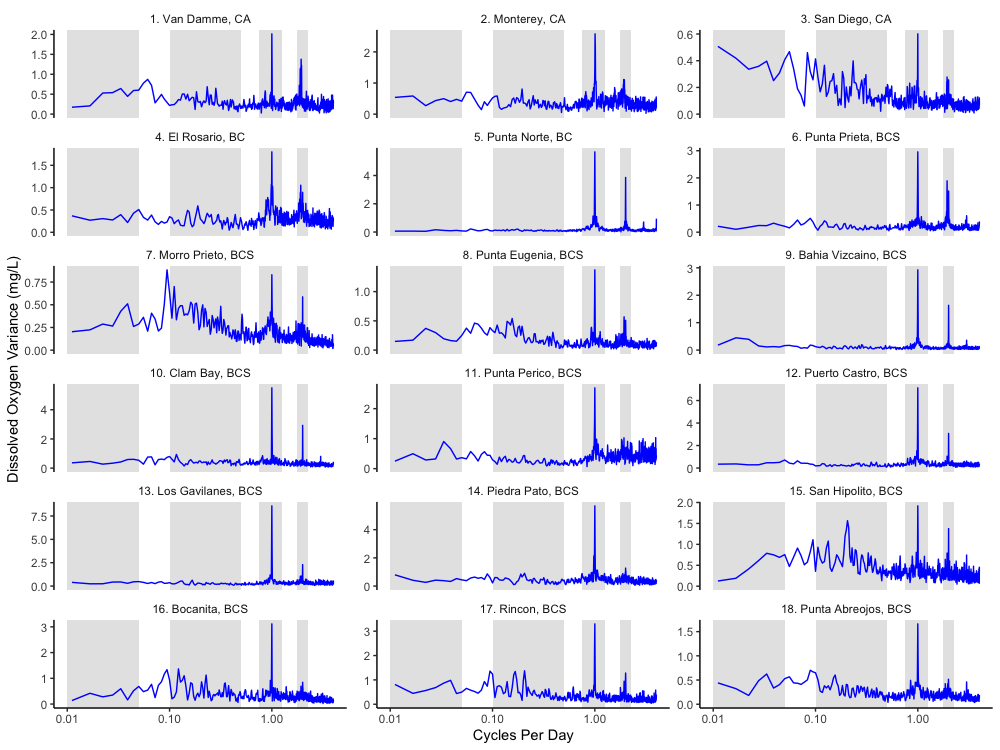


Figure S6. Variance of temperature (red bars) and dissolved oxygen (blue bars), integrated over seasonal (0.01-0.05 cycles per day; cpd), synoptic (﻿scales of mesoscale variability and atmospheric weather patterns; 0.1-0.5 cpd), diurnal (0.75 – 1.25 cpd), and semidiurnal (1.75 – 2.25 cpd) bands. Note the site-specific differences in the range of variance (y-axis).


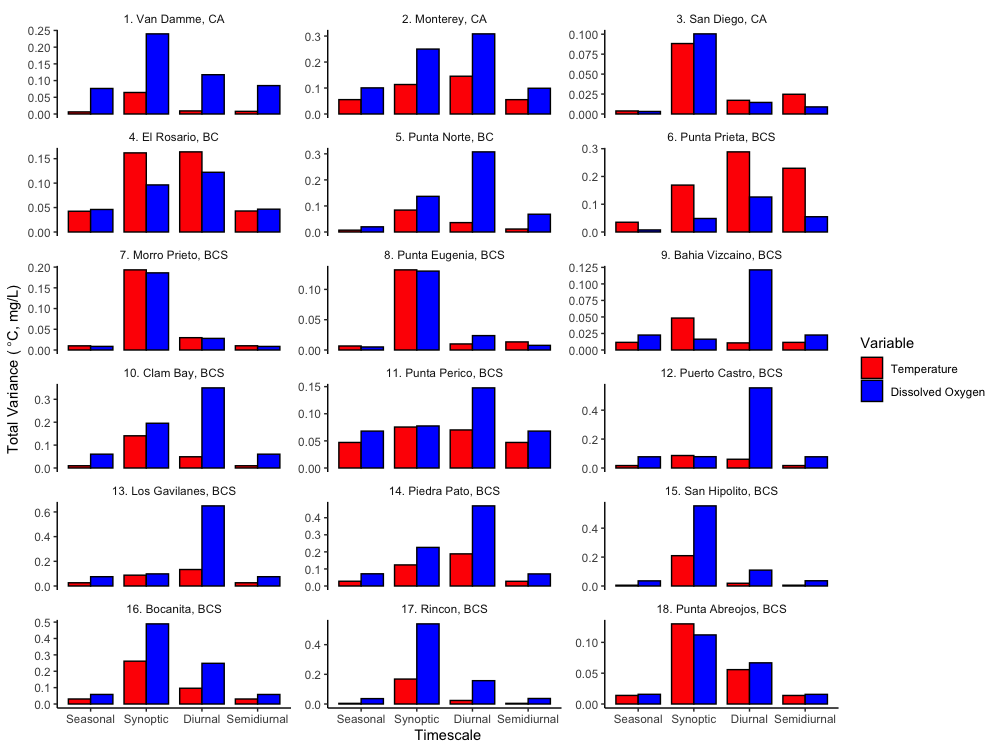

Supplement: Supplementary file 1 — Supplementary Information. [file 41598_2021_89928_MOESM1_ESM.docx]
